# Incidence of depressive symptoms among sexually abused children in Kenya

**DOI:** 10.1186/s13034-018-0247-y

**Published:** 2018-07-30

**Authors:** Teresia Mutavi, Anne Obondo, Donald Kokonya, Lincoln Khasakhala, Anne Mbwayo, Francis Njiri, Muthoni Mathai

**Affiliations:** 1 Department of Psychiatry, School of Medicine, College of Health Sciences, University of Nairobi, P.O. Box 74-00519, Mlolongo, Nairobi, Kenya; 2 Department of Behavioural Sciences & Community Health, School of Medicine, Masinde Muliro University of Science and Technology, Kakamega, Kenya

**Keywords:** Children, Depressive symptoms, Sexual abuse, Kenya

## Abstract

**Background:**

Children who experience sexual abuse undergo various negative psychosocial outcomes such as depressive symptoms. Unfortunately, not many studies have been conducted on the incidence of depressive symptoms among sexually abused children in Kenya. This study sought to ascertain the incidence of depressive symptoms among children who have experienced sexual abuse in Kenya.

**Methods:**

This was a longitudinal study design. It was conducted at Kenyatta National Teaching and Referral Hospital and Nairobi Women’s Hospitals in Kenya. One hundred and ninety-one children who had experienced sexual abuse and their parents/legal guardians were invited to participate in the study. The study administered the Becks Depression Inventory and the Child Depression Inventory to the children.

**Results:**

The incidence of depressive symptoms after 1 month of sexual abuse revealed that amongst children who were below 16 years old, 14.6% had minimal-mild depressive symptoms while 85.4% had moderate-severe depressive symptoms. In comparison, children who were 16 years or older, 6.4% had minimal-mild depressive symptoms while 93.6% had moderate-severe depressive symptoms. Children below 16 years old whose parents were separated were found to have depressive symptoms (p < 0.001) as well as those who were presented early for medical care (p < 0.004), while children aged 16 years and above who were abused by strangers were more likely to have depressive symptoms (p < 0.024) and those who were not attending school (p < 0.002).

**Conclusion:**

Sexual abuse of children is world-wide and the Kenyan situation is comparable. Being the victim of sexual abuse as a child has major psychological and emotional sequlae which need to be addressed in Kenya. Children who experience sexual abuse have very high incidence of developing depressive symptoms. All the sexually abused children studied suffered from depressive symptoms and a large majority suffered from major depressive symptoms that should be promptly and effectively addressed to ameliorate psychological suffering among children.

## Background

Violence against children is a widespread global problem, causing detrimental health and social outcomes to the child, the family and society [1]. The global estimated prevalence of sexual abuse among girls ranged from 7 to 36% and 5 to 10% among boys [2]. Globally, an estimated one billion children were exposed to violence during the year 2014 compared to two billion children in 2013 who experienced either physical, emotional or sexual violence [3]. Children were found to be affected by sexual abuse across different age groups globally [4]. Sexual abuse had been common among girls than boys and girls were at a higher risk of re-exposure than boys [5].

Accordingly, the odds of having been exposed to sexual abuse had been shown to be greater among girls than boys (OR 1.29; 95% CI 1.2–1.48; p < 0.001) [6]. In Ethiopia, among 267 children who were treated at two hospitals after experiencing sexual abuse, 75.7% were girls and 24.3% were boys [7]. A review paper of African countries in 2014 reported that the odds for girls experiencing sexual abuse was higher than boys (OR 1.85–3.85 and p < 0.01) [8]. In the year 2011, a Tanzanian study on children aged 13–17 years old found out that nearly two-thirds (63.9%) of the girls and slightly over a third (38.7%) of the boys had experienced sexual abuse [9]. In a Ugandan study of 2014, more girls than boys experienced sexual abuse [*X*^2^(1407) = 12.44; *p *< 0.0005] [10]. The prevalence of sexual abuse among children under 18 years of age in Kenya over a period of 5 years (2007–2013) reported that 11.8% of the girls and 3.6% of the boys had been exposed to sexual abuse [11]. An earlier study during the period 2006–2009 found out that 28.3% of the sexual abuse cases among children assessed and treated at Kenyatta National Hospital (KNH), Nairobi Kenya were young and below 18 years of age [12]. Psychosocial outcomes associated with sexual abuse included depression [13], post traumatic-stress disorder (PTSD) and declining school grades among many other negative outcomes [14]. The Ugandan study of 2013 found out that among 1587 children who had experienced childhood traumas had a higher risk of developing depressive symptoms [15]. In the Ethiopian study of 2009, students who reported experiencing different forms of sexual abuse were nearly twice as likely to be classified as having moderate depressive symptoms compared to those who had not been sexually abused [16]. Similarly, in the study of 2015 among 1456 boys in Kenya aged 13–24 years who had experienced sexual abuse, it was found out that 90% of them had depressive symptoms and 14.8% had experienced sexual abuse before the age of 18 years [11]. Another study of 2015 on adolescents aged 13–18 years in Kenya found out that 80% of those who had experienced sexual abuse and other traumatic events had varying degrees of depressive symptoms and 89.2% of them had experienced difficulties in academic performance [17]. This study sought to determine the incidence of depressive symptoms among Kenyan children who had been sexually abused. The children were followed up for a period of 1 year in order to highlight the importance of psychosocial support and to improve the mental health of these children.

## Methods

This paper was part of a Kenyan longitudinal study on the psychosocial outcomes associated with sexual abuse among children aged 7–17 years who were treated at the Kenyatta National Hospital (KNH), the National Teaching and Referral Hospital. The study was also conducted at a specialised private Gender-Based Violence (GBV) hospital, the Nairobi Women’s Hospital in Nairobi, Kenya [18]. The two are the leading and specialized hospitals in (GBV) in Kenya, based in Nairobi City. Sexually abused children are medically cared for at the two hospitals with 24-h service at the Gender Based Violence Recovery Centres (GBVRC) or at the Accident & Emergency (A&E) departments [19]. Physical and mental state assessments are conducted by trained medical officers and psychiatrists respectively and data secured confidentially [20]. post exposure prophylactic, emergency contraception, treatment for sexually transmitted infections (STI) and human immunodeficiency virus (HIV) are routinely offered to the survivors who fill in locator forms [21]. The KNH, GBV service centre treats and supports an average of 6 survivors per day while the GBV service centre at the Nairobi Women’s Hospital’s (NWH) treats and supports an average of 10 survivors. Both centres provide a one stop patient management. This includes provision of emergency post rape medical care, collection and preservation of forensic evidence, Legal Aid, medical intervention, creation of awareness about GBV, trauma counselling, outreach programmes and establishment of support groups for survivors of GBV [22].

This study was approved by the Ethical and Research Committee (ERC) of the KNH/University of Nairobi [23]. The study population comprised of GBV survivors aged 7–17 years. A minimum sample size was set [N = 191] at baseline including an assumed attrition rate of 20% [24, 25]. Four months later, at the first follow up, the attrition rate was 6%, 8 months later it was 6 and 4% at the end of the study period. The cumulative attrition rate was 14% well within the 20% anticipated attrition rate. The attrition was attributed to relocation of the family to the rural areas while others did not return for follow up despite several telephone reminders. The study participants were recruited into the study when they were presented by their care takers for medical and psychosocial care at the two study sites. They were recruited using systematic and purposive (by gender) sampling techniques. After a child had been randomly selected to participate in this study after 1-month exposure to the sexual abuse incidence, the parents/legal guardians were approached to give informed consent to participate in this study and assent from the children. The children were put on the standard prophylactic and therapeutic treatments regimes, usually provided at the GBVR centres in both hospitals [18]. This included Post exposure Prophylaxis (PeP) against HIV and contraceptive prophylaxis (CP), treatment of physical injuries and psychosocial care in the hospitals and or referred to legal services providers. The study participants who were found to be in need of medical care were referred to physicians at the two centres.

A locally designed questionnaire on socio-demographic and sexual abuse profile was administered to the GBV survivors aged 7–17 years. The Beck Depression Inventory (BDI) was used for screening depressive symptoms among children aged 16–17 years because adolescents were considered as mature youth in the study and the Children’s Depression Inventory (CDI) was used for screening depressive symptoms in the children aged 7–15.5 years [18]. On the CDI tool, minimal and mild depressive symptoms were regarded as insignificant and moderate and severe depressive symptoms were regarded as pathological [26]. The CDI used was a downward extension of the BDI. Higher scores on the BDI denoted greater severe depressive symptoms. The CDI had high correlational reliability for depressive symptoms with a Cronbach’s alpha value of 0.86 for a psychiatric sample and 0.82 for a paediatric medical sample. The total score was the sum of all the separate item scores. Reliability and validity had been established over many years of empirical research. The CDI had been successfully used in the Kenyan children’s population [27]. It had also been validated in the Sub Saharan African countries in Rwanda [28], Tanzania [29] and in Malawi [30]. The BDI had been adequately adapted to the Kenyan population [31]. The BDI and the CDI had been translated into Kiswahili versions, a *lingua franca* widely spoken in Kenya, Eastern and Central Africa [29, 30].

Data was collected over a 1-year period at follow up intervals of 4 months. Completed questionnaires were safely and securely stored prior to entry into computer excel sheets. Descriptive statistics were analysed, interpreted and displayed while inferential statistics were statistically analysed using SPSS Version 21. The level of statistical significance was set at 0.05 (*p *< 0.05) with a 95% confidence interval (CI). Bivariate logistic regression analysis and analysis of variance were used to determine associations among variables.

## Results

### Socio-demographics

One hundred ninety-one (N = 191) sexually abused children were recruited into this study, of whom 12.0% were males and 88.0% were females, giving a male: female ratio of 1:7. The mean and median age were equal at 13 years. The youngest study participant was 7 and oldest the was 17 years of age. The majority (35.1%) of the study participants were young in their mid-adolescence at (13–15 years) of age. Almost a quarter (24.6%) were in their older (16–17 years) childhood ages while slightly less than a quarter (23%) were aged (10–12 years) early adolescence and 17.3% were below adolescence (7–9 years). A statistically significant proportion (96.8%) of the study participants attended school and only 3.2% did not attend school. The parents of over three quarter (75.4%) of the participants were alive while 17.3% of the children had living mothers but no fathers. Less than five percent (4.2%) had fathers only and 3.1% were parentless (orphaned). Slightly less than three quarters (72.2%) of the parents were married, 19.4% were separated or divorced and 8.4% were of single parentage. Nearly a third (30.4%) of the parents/legal guardians earned less than US$1/day but the majority (37.2%) earned US$1/day, leaving only 29.3% living above the poverty line with a monthly income of more than US$1/day and 3.1% earned more than US$2 a day.

### Sexual abuse

Slightly over a half 97 (50.7%) were abused by acquaintances, 80 (41.8%) by a stranger, 3 (1.7%) by a care giver, 9 (4.8%) by biological parent and 2 (1%) by foster parent. Majority 170 (89%) of the participants experienced vaginal anal penetration, 11 (5.8%) experienced touching of the genital, 10 (5.2%) experienced non-genital contact. Majority 172 (90.1%) were made to touch the genitals of the perpetrator, 15 (7.8%) were not made to perform any act and 4 (2.1%) were made to do oral copulation. Ninety two (48.1%) of the abuse incidence happened once, 38 (19.9%) of the abuse happened twice, 30 (15.7%) of the abuse incidence happened three times, 16 (8.4%) of the abuse incidence happened four times and lastly 15 (6.7%) of the abuse incidence happened more than four times. Most 129 (67.9%) the abuse had taken place over a month ago, 42 (22.1%) took place over the past few days, 5 (2.1%) took place over a week ago and 15 (7.9%) had taken place over years ago.

### Incidence of depressive symptoms

Depressive symptoms were assessed using CDI at baseline for children below 16 years old and Becks Depression Inventory for children over 16 years. Almost three quarters (3/4) of the study participants (74.3%) experienced moderate depressive symptoms and 11.1% experienced severe depressive symptoms. It was therefore, noted that approximately 85.4% of the sexually abused children in Kenya experienced major depressive symptoms. This meant that sexually abused children in Kenya, suffered major depressive symptoms (T score > 60, equivalent of > 84th percentile) that warranted special mention and attention. Only 14.6% of the children did not experience major depressive symptoms and 3.5% of them suffered from minimal depressive symptoms while 11.1% experienced mild depressive symptoms. During the first follow up, 16% of the study participants had minimal depressive symptoms 81.9% had mild depressive symptoms, 2.1% had moderate depressive symptoms while none had severe depressive symptoms. By extension, during the second follow, up 92% of the children had minimal depressive symptoms and there were no major depressive symptoms after the first follow up (Table 1).

**Table 1:** Incidence of depressive symptoms (N = 166)

	Baseline		Follow ups					
			1		2		3	
	N	%	N	%	n	%	n	%
CDI								
Minimal < 10	5	3.5	23	16.0	127	92.0	132	100.0
Mild, 11–16	16	11.1	118	81.9	11	8.0	0	0.0
Moderate, 17–27	107	74.3	3	2.1	0	0.0	0	0.0
Severe > 28	16	11.1	0	0.0	0	0.0	0	0.0
Totals	144	100	144	100	138	100	132	100
BDI								
Minimal, 0–9	0	0.0	0	0.0	26	74.3	27	79.4
Mild, 10–16	3	6.4	14	38.9	9	25.7	7	20.6
Moderate, 17–29	27	57.4	22	61.1	0	0.0	0	0.0
Severe, 30–63	17	36.2	0	0.0	0	0.0	0	0.0
Totals	47	100	36	100	35	100	34	100

This findings study showed that there was a direct relationship between sexual abuse and depressive symptoms. The therapeutic interventions at the GBVRCs in Kenya were rapid and effective as shown by the absence of depressive symptoms, 4 months after the first follow up session and all the children had achieved remission (Table 1).

Children below 16 years of age whose parents were separated were found to be vulnerable to depressive symptoms (*p *< 0.001) as well as those under the of care givers (*p *< 0.0001). The older children aged 16 years and above who were not attending school were found to be more likely to have depressive symptoms (*p *< 0.002), as were children who lived with their fathers (*p *< 0.0018) or were under the care of good Samaritans (*p *< 0.0001) (Table 2).

**Table 2:** Depressive symptoms in relation to socio demographic characteristics (N191)

	CDI score		BDI score	
	Mean	p-value	Mean	p-value
Gender				
Male	22.3	0.624	29.0	0.867
Female	21.5		27.7	
Attending school				
Yes	21.5	0.667	27.1	0.002
No	19.5		43.0	
School level				
Primary	21.7	0.579	26.2	0.190
Secondary	20.2		29.2	
Have parents				
Both mother and father	22.0	0.575	26.7	0.018
Only mother	20.3		27.8	
Only father	20.1		49.0	
None	26.0		31.0	
Parents marital status				
Married	20.7	0.001	26.3	0.141
Separated	25.9		29.8	
Divorced	18.5		31.0	
Others	19.1		33.1	
Who do you live with				
Good samaritan	20.0	< 0.0001	49.0	< 0.0001
Care giver	20.8		25.6	
Guardian	27.7		32.7	
Others	.		41.0	

The female children aged 16–17 years experienced moderate depressive symptoms (mean score = 27.7) while the male experienced severe depressive symptoms (mean score = 29.0) on the BDI II scale. The out of school children experienced severe depressive symptoms (mean score = 43) while those in school experienced moderate depressive symptoms (mean score = 27.1) but those in secondary schools experienced severe depressive symptoms (mean score = 29) compared to those in primary school who experienced moderate depressive symptoms (mean score 26.2). Being raised by the father alone (mean score = 49) or having no parent (mean score = 31) were risk factors for severe depressive symptoms but having both parents (mean score = 26.7) or a mother only (mean score 27.8) led to moderate depressive symptoms. Children whose parents were marriage experienced moderate depressive symptoms (mean score = 26.3) compared to divorced (mean score = 31.0, separated (mean score = .29.8 and others (mean score = 33.1) all of whom experienced severe depressive symptoms. Children under the care of good Samaritans (mean score = 49), guardian (mean score. = 27.7) and others (mean score = 41.0) were risk factors for severe depressive symptoms while children under care givers (mean score 25.6) experienced moderate depressive symptoms.

Though statistically insignificant (p < 0.624), both male and female younger children aged 7–15.5 years experienced severe depressive symptoms (mean score 22.3 and 21.5 respectively). On the CDI I depressive symptoms scale, the children out of school experienced mild depressive symptoms (mean score = 19.5) compared to moderate depressive symptoms experienced by those in school (man score = 21.5). Both primary and secondary school children aged 7–15.5 years experienced moderate depressive symptoms (mean score—21.7 and 20.2 respectively). Whether the parents of the children were alive or not, all the children experienced moderate depressive symptoms with high mean scores (both = 22.0), mother only (mean score = 20.3), father only (mean score = 20.1) and none (mean score = 26.0). Whereas the children of divorced and other status of parenthood experienced mild depressive symptoms (mean score = 18.5 and 19.1 respectively), the children of the married parents experienced moderate depressive symptoms (mean score = 20.7) and so to the separated (mean score = 25.9). Living with a good Samaritan (mean score = 20.0), care givers (mean score = 20.8) and guardians (mean score = 27.7) were risk factors for moderate and severe depressive symptoms respectively (Table 2).

Sexually abused young children below 16 years of age, in spite of receiving comprehensive and specialized medical care early were more likely to be depressed (*p *< 0.004), while those above 16 years of age who were abused by strangers were more likely to be depressed (*p *< 0.024). Furthermore, children who were forced to touch the perpetrators’ genitals (*p *< 0.011) and those who were taken to the police stations (*p *< 0.032) were more likely to have depressive symptoms (Table 3).

**Table 3:** Depressive symptoms in relation to sexual abuse profile (N = 191)

	CDI score		BDI score	
	Mean	p value	Mean	*p* value
Relationship to perpetrator				
Stranger	21.4	0.199	29.1	0.024
Acquaintance	21.3		26.1	
Non-parental care giver	18.0		15.0	
Biological parent	26.1		41.0	
Perpetrator acts				
Vagina anal penetration	21.3	0.109	27.4	0.230
Touching the genitals	26.5		40.0	
Non-genital contact	18.0		–	
What perpetrator made victim do				
Nothing	19.1	0.458	25.7	0.011
Touching genitals	21.8		49.0	
Oral copulation	20.0		–	
Others	–		27.5	
Frequency of abuse				
Once	20.4	0.129	27.6	0.229
Twice	22.7		27.4	
Three times	22.7		31.0	
Four times	24.9		20.0	
More than four times	20.9		40.0	
How long the abuse taken place				
Days	20.6	0.748	28.0	0.875
Weeks	21.7		22.0	
Months	21.8		27.2	
Years	23.1		27.5	
First place to be taken				
Hospital	21.9	0.469	26.9	0.032
Chief’s camp	–		21.0	
Police station	20.4		33.2	
Other	18.0		41.0	
How long before receiving medical care				
Within 1 h	21.9	0.004	26.9	0.223
Within 2 h	25.7		31.0	
Within 12 h	22.7		25.0	
Within 48 h	12.0		24.0	
Within 72 h	21.7		–	
After 72 h	17.6		35.0	

The 7–15.5-year-old children experienced severe depressive symptoms perpetrated by their biological parents (mean score = 26.1), moderate depressive symptoms (mean scores = 21.4 and 21.3) perpetrated by strangers and acquaintances respectively. However, non-parental care givers caused mild depressive symptoms (mean score = 18.0) on the CDI I scale. On the BDI II scale among the 16–17 year old children, biological parents and strangers perpetrated severe depressive symptoms (mean score = 41 and 29.1 respectively), acquaintances caused moderate depressive symptoms (mean score = 26.1) while non-parental care giver was associated with mild depressive symptoms (mean score = 15.0). There was strong association between sexual abuse and depressive symptoms among the older 16–17 years (*p *< 0.024) but this was not the case with the younger children aged 7–15.5 years (*p *< 0.199). As to whether there was a relationship between depressive symptoms and the acts of the perpetrator, there was no statistical significance in both the younger and the older age groups (*p *< 0.109 and 0.230 respectively). Touching the minors’ genitals 7–15.5 years (mean score = 26.5) evoked severe depressive symptoms to the highest level as well as the 16–17 years old (mean score = 40.0) on the BDI II scale. Between the 7–15.5 and the 16–17 year old, the victims experienced severe depressive symptoms (mean score = 26.5 and 49.0 respectively) when compelled to touch the perpetrators’ genitals compared to the rest, oral copulation (mean score = 20.0) for the younger children and none (mean score = 19.1) and others (mean score = 27.5) and none (mean score = 25.7) for the older children. The modal frequency of sexual abuse was four (× 4) times among the younger children aged 7–15.5 years old (mean score = 24.9) causing moderate depressive symptoms, though it was not statistically significant (*p *< 0.129). Among the older children (16–17 years), the modal frequency of sexual abuse was > four (× 4) times, causing severe depressive symptoms (mean score = 40), also not statistically significant (*p *< 0.229). There was moderate depressive symptoms in relation to the severity of sexual abuse with regard to the duration of abuse, ranging from days to years (mean score = 20.6–23.1) which produced a pattern showing that the longer the younger children were abused, the severer the depressive symptoms they experienced. In the case of the older children (16–17 years), all of them experienced moderate depressive symptoms (mean score = 28–22) and the abuse taking place for days was the highest (mean score = 28) which caused the upper end of moderate depressive symptoms. The findings in both categories of children were statistically insignificant (*p *< 0.748 and 0.875 respectively). Following the sexual abuse, the young children were mostly taken to hospitals (mean score = 21.9) followed by police stations (mean score = 20.4) and other places (mean score = 18.0). The older children were largely taken to unknown places (mean score = 41.0) followed by police stations (mean score = 33.2), then hospitals (mean score = 26.9) and Chiefs’ camps (mean score = 21.0). Within a range of 1–72 h and above, the young victims accessed healthcare within significant short periods (*p *< 0.004), particularly 2 h (mean score = 25) while it took longer than 72 h for the older children (p < 0.223) to be taken for healthcare (mean score = 35) (Table 3).

## Discussion

Sexual abuse in Kenya was widespread and it caused depressive symptoms among children producing similar trends to international findings (1, 3). The mean age in this study was consistent with those of other studies conducted in Kenya [32, 33]. Female children were seven times (× 7) at risk of sexual abuse in Kenya compared to the males in the mid adolescence age bracket and sexual abuse started in their early ages, just like elsewhere worldwide [1, 2, 5, 6, 10, 32, 33]. These Kenyan findings supported those of previous studies on sexual abuse among children [32, 33] as well as International studies [3, 5, 34]. Almost all cases of sexual abuse were in school, implying that either literacy rate was high and or schooling was a predisposing factor and a large majority were under both parental care in marriage [9, 11]. Most of the victims hailed from families which lived below poverty line and over four-fifths (4/5) of the children experienced severe depressive symptoms beyond the third percentile, quite comparable to studies in the USA [35, 36]. Timely and professional intervention showed that the children could recover fast to full remission within 4 months of the traumatic experience [7, 27]. The findings in this study showed that there was a temporal relationship between sexual abuse and depressive symptoms [11, 13, 16, 17, 37, 38]. These findings were comparable to those in Ethiopia where findings showed that the sexually abused adolescents were twice more likely to experience depressive symptoms than non-abused adolescents [11, 13, 16]. There was vulnerability to sexual abuse among children when parents were divorced or separated and when the children were left in the hands of care givers [38]. The high prevalence [2, 11, 13] and incidence of depressive symptoms particularly among the older children were associated with single parenthood and schooling (almost all the children were in school) but the out of school children experienced severe depressive symptoms that was comparable to other findings [11, 13, 16, 17, 32, 33]. It did not matter whether the parents of the children were alive or not, they all had similar experiences which were comparable with other studies done in Kenya [32]. The younger the children who were sexually abused and those abused by strangers, the severe the depressive symptoms among them [25, 26]. Biological parents who sexually abused their children were found to experience severe depressive symptoms compared to those abused by strangers and there was association between acts of sexual abuse and depressive symptoms [32, 33]. This study also showed that sexual abuse including repeated sexual abuse was common amongst children in Kenya, with associated depressive symptoms which was similar to other studies [5]. Children who were first taken to the hospital within 3 days experienced less depressive symptoms than those who were taken to police stations or elsewhere, a finding similar to that in another study in Kenya [27]. Based on this finding, given that children promptly taken to hospital have less depressive symptoms, it suggests that prompt interventions may have a role in ameliorating depressive symptoms. There appears to be an association between sexual abuse and depressive symptoms, and that therapeutic interventions at the GBVRCs in Kenya are temporally associated with reduction in depressive symptoms, with more rapid intervention associated with less depressive symptoms.

## Conclusion

Sexual abuse of children is world-wide and the Kenyan situation is comparable. The act has major psychological and emotional outcomes that need to be addressed in Kenya. Children who experienced sexual abuse had high likelihood of developing depressive symptoms. All children in this study suffered from depressive symptoms 1 month after sexual abuse and that should be urgently addressed to reduce psychological suffering among children in Kenya. Screening and immediate provision of treatment after sexual abuse among children is important to prevent mental health problems among children for the improvement of their psychosocial functioning. Parents whose children have experienced sexual abuse need psychosocial support to enable their children cope with the trauma, thereby quickly facilitating their recovery process.

## Abbreviations

SVAC: sexual abuse against children
OR: odds ratio
GBVRC: Gender Based Violence Recovery Centres
BDI: Becks Depression Inventory
CDI: Children’s Depression Inventory
ERC: Ethical and Research Committee
